# The Emerging Role of Dimethyl Fumarate in Alzheimer’s Disease—A Systematic Review of Available Preclinical Studies

**DOI:** 10.3390/ijms27104227

**Published:** 2026-05-09

**Authors:** Maria Mouaimi, Athanasios Metaxas, Malamati Kourti

**Affiliations:** Department of Life Sciences, School of Sciences, European University Cyprus, Nicosia 2408, Cyprus; mariamouaimi01@gmail.com (M.M.); a.metaxas@euc.ac.cy (A.M.)

**Keywords:** dimethyl fumarate (DMF), Alzheimer’s disease, oxidative stress, Nrf2, antioxidant, neuroprotection

## Abstract

Dimethyl fumarate (DMF), a fumaric acid ester, is approved for psoriasis and multiple sclerosis due to its antioxidant and anti-inflammatory properties mediated via Nrf2 activation. Nrf2 regulates genes that protect cells from oxidative stress, a key factor in neurodegenerative diseases such as Alzheimer’s disease (AD), which is characterized by amyloid-β and tau accumulation and lipid peroxidation. This systematic review aimed to evaluate preclinical evidence for DMF as a potential therapeutic agent in AD models through Nrf2 activation. A comprehensive literature search of PubMed and Scopus (last search: December 2025) identified in vitro, in vivo, and combined preclinical studies assessing DMF in AD models. Studies were screened using predefined inclusion and exclusion criteria, and methodological quality was assessed using established tools. Results were synthesized narratively. Eighteen studies were ultimately included in the analysis. Across the included studies, DMF consistently activated the Nrf2 pathway, enhancing antioxidant and anti-inflammatory gene expression. DMF treatment reduced amyloid-β and tau protein levels, mitigated oxidative stress, and improved cognitive performance in animal models. However, the evidence is limited by heterogeneity in experimental models and methodological variability. In conclusion, preclinical evidence suggests DMF is a promising candidate for AD treatment by targeting oxidative stress and neuroinflammation via Nrf2 activation. Further preclinical studies, particularly on ferroptosis mechanisms, and well-designed clinical studies are warranted to clarify its full therapeutic potential. This review was not registered and the authors received no funding.

## 1. Introduction

### 1.1. Pathophysiology and Therapeutic Challenges of Alzheimer’s Disease

Population aging worldwide has led to a substantial increase in neurodegenerative diseases, with Alzheimer’s disease (AD) representing the leading cause of dementia worldwide [1]. AD is a progressive and ultimately fatal neurodegenerative disorder. It is characterized by memory impairment, cognitive decline, and loss of functional independence [2,3]. Given its growing global burden, the World Health Organization has designated dementia, including AD, as a public health priority [4].

Epidemiologically, AD accounts for the majority of dementia cases and affects approximately 10–30% of individuals over 65 years of age [4,5,6]. It is among the leading causes of death in elderly populations in both the United States and Australia. Women exhibit a higher lifetime risk than men, potentially reflecting sex-specific differences in risk profiles and longevity [7]. Globally, more than 35 million individuals are affected, and projections indicate a doubling of cases within the next two decades [8]. This escalating burden highlights the urgent need for disease-modifying interventions.

**Figure 1:**
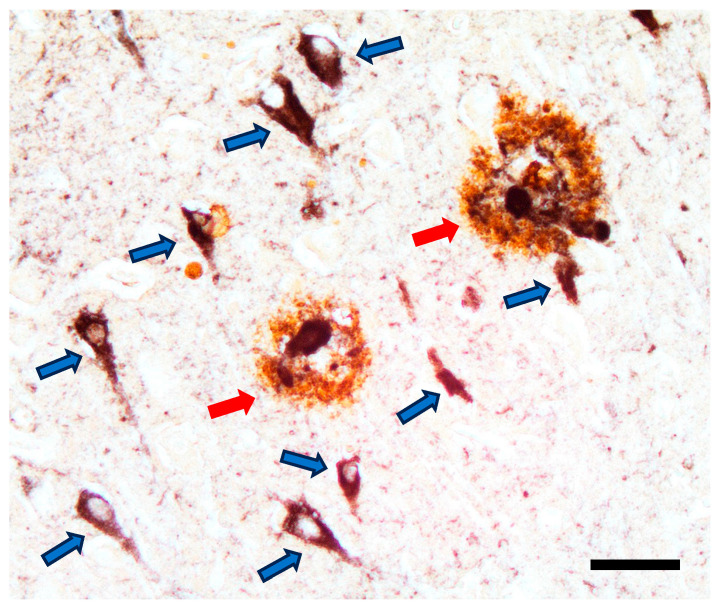
Hallmark AD pathologies. Dual immunohistochemical stain using antibodies to Aβ (brown) and tau proteins (black), depicting the two defining proteopathies of AD. Abnormal, hyperphosphorylated tau occurs in neuronal cell bodies, in fine neuronal processes throughout the neuropil, and in swollen neurites within the plaques. Scale bar: 50 μm. Red arrows indicate β-amyloid plaques and blue arrows indicate tau-positive neurofibrillary tangles. Image adapted from ‘Abeta-Tau-AD.tif’, Wikimedia Commons, licensed under Creative Commons Attribution-ShareAlike 4.0 (CC BY-SA 4.0).

Several theories have been proposed to explain the pathogenesis of AD. These include the amyloid cascade hypothesis, the tau hypothesis, and mechanisms involving oxidative stress and neuroinflammation. Neuropathologically, AD is defined by extracellular deposition of amyloid-β (Aβ) plaques and intracellular accumulation of hyperphosphorylated tau protein in the form of neurofibrillary tangles (NFTs) [6] (Figure 1). The amyloid cascade hypothesis suggests that abnormal Aβ production and aggregation play a central initiating role, while the tau hypothesis emphasizes the contribution of tau hyperphosphorylation to neuronal dysfunction. Beyond these classical hallmarks, multiple interrelated mechanisms contribute to disease onset and progression, including oxidative stress, mitochondrial dysfunction, impaired cerebral glucose metabolism, vascular abnormalities, and chronic neuroinflammation [9]. Increasing evidence suggests that AD is a multifactorial disorder involving complex interactions between protein aggregation, redox imbalance, and immune dysregulation [10].

Oxidative stress plays a central role in AD pathogenesis. Reactive oxygen species (ROS), such as superoxide anion radical (O_2_^•−^) and hydroxyl radical (HO^•^), are generated during normal cellular metabolism but become detrimental when antioxidant defences are impaired [11]. The brain is particularly vulnerable to oxidative damage due to its high oxygen consumption, which is approximately 20% of total body oxygen use, abundant lipid content, and relatively limited regenerative capacity [12,13]. In AD, excessive ROS production contributes to lipid peroxidation, mitochondrial dysfunction, and disruption of synaptic plasticity, including long-term potentiation [14]. Oxidative imbalance further promotes Aβ aggregation and tau pathology, creating a self-amplifying cycle of neurodegeneration [13].

Currently approved pharmacological treatments for AD, including cholinesterase inhibitors (e.g., donepezil, galantamine) and the NMDA receptor antagonist memantine, provide only symptomatic relief and do not halt neurodegeneration [15,16]. In recent years, therapeutic strategies have increasingly focused on targeting the core pathological features of AD, particularly Aβ and tau protein aggregation [17]. However, clinical efficacy remains limited, particularly once significant neuronal loss has occurred [18].

Passive immunotherapy with monoclonal antibodies (mAbs) directed against pathogenic Aβ species represents a major advance and aims to promote amyloid clearance through mechanisms such as microglial-mediated phagocytosis and neutralization of toxic Aβ aggregates. First-generation anti-Aβ mAbs largely failed to demonstrate clinical efficacy [19]. However, second-generation mAbs such as lecanemab, donanemab, and aducanumab have shown the ability to reduce amyloid plaque burden and, in some cases, slow cognitive decline in early AD patients [20]. Lecanemab (Leqembi^®^) preferentially targets soluble aggregated Aβ protofibrils and has demonstrated significant reduction in amyloid load and slowed clinical decline in the Phase III CLARITY-AD trial, leading to full U.S. Food and Drug Administration (FDA) approval in 2023 [21]. Mechanistically, lecanemab exhibits high selectivity for soluble Aβ protofibrils over monomers and fibrils, facilitating their clearance and reducing downstream neurotoxicity [22]. Donanemab (Kisunla™), which targets an N-terminal pyroglutamate Aβ epitope, has similarly shown plaque clearance and modest slowing of cognitive and functional decline in large Phase III studies, receiving FDA approval in 2024 [23]. Aducanumab (Aduhelm^®^) received accelerated approval based on its plaque-lowering effects, though its clinical benefit remains debated [24,25,26,27]. Despite these advances, the clinical benefit of anti-Aβ treatment is often modest and accompanied by risks such as amyloid-related imaging abnormalities (ARIAs), including brain oedema and microhaemorrhages, particularly in APOE ε4 carriers [28,29].

Parallel efforts have targeted tau pathology, which correlates more closely with cognitive impairment than amyloid load [30]. Several anti-tau antibodies designed to inhibit tau aggregation and seeding are in clinical development. For example, E2814 (Etalanetug) is a monoclonal antibody targeting the tau microtubule-binding domain, currently under evaluation in Phase I/II trials alone (NCT04971733) or in combination with lecanemab (NCT06602258), with outcomes expected through 2027 [31,32]. Other tau-directed approaches, such as posdinemab, aim to bind specific phosphorylated tau epitopes, though early studies have yielded mixed results [33]. Some anti-tau candidates have been discontinued after failing to demonstrate efficacy in Phase II trials, highlighting the challenges of targeting tau pathology directly [34]. Beyond conventional mAbs, emerging strategies include bispecific antibodies against multiple pathological targets, vaccines to induce active immunity against tau, and next-generation drugs optimized for blood–brain barrier penetration. Although anti-amyloid and anti-tau immunotherapies represent significant progress toward disease-modifying treatment, clinical efficacy remains limited, particularly once significant neuronal loss has occurred, highlighting the need for novel strategies that address the multifactorial pathophysiology of AD, including oxidative stress modulation and neuroinflammation control [18].

### 1.2. The Nrf2 Pathway in Oxidative Stress and Neuroprotection

Maintenance of redox homeostasis is essential for cellular integrity. Nuclear factor erythroid 2-related factor 2 (Nrf2), encoded by the *NFE2L2* gene, is a master transcriptional regulator of antioxidant and cytoprotective responses [35,36,37]. Since its identification in 1994, Nrf2 has been recognized as a central mediator of cellular defence against oxidative and electrophilic stress.

Nrf2 regulates the expression of genes involved in redox balance, detoxification, mitochondrial function, iron metabolism, and DNA damage responses [35,38]. Structurally, Nrf2 contains seven conserved Nrf2-ECH (NEH) domains that coordinate its regulatory activity. The NEH1 domain enables heterodimerization with small MAF (sMAF) proteins and then binding to antioxidant response elements (AREs), DNA enhancer sequences that drive transcription of antioxidant and cytoprotective genes [37,39]. The NEH2 domain mediates interaction with Kelch-like ECH-associated protein 1 (Keap1), the main negative regulator of Nrf2.

**Figure 2:**
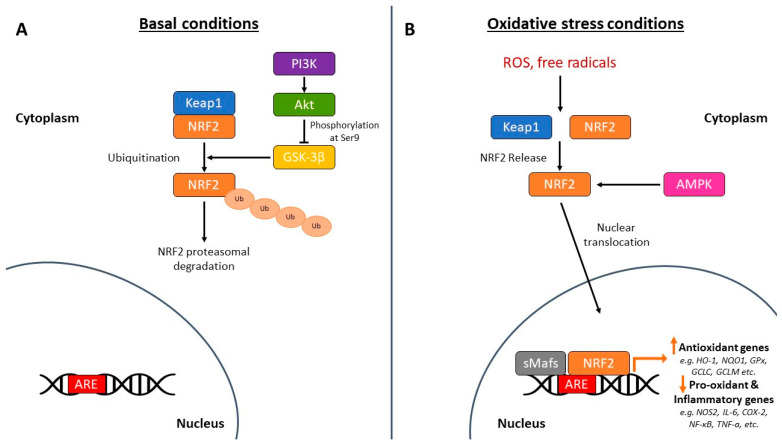
Mechanism of Nrf2 under basal and oxidative stress conditions. (**A**) Under basal conditions, Nrf2 is sequestered by Keap1 in the cytoplasm, leading to ubiquitination and proteasomal degradation. GSK-3β also promotes Nrf2 degradation, while PI3K/AKT-mediated inhibition of GSK-3β stabilizes Nrf2. (**B**) Under oxidative stress, Nrf2 dissociates from Keap1, translocates to the nucleus, dimerizes with sMafs, and binds ARE sequences to induce antioxidant genes (e.g., *HO-1, NQO1*, *GPX*, *GCLC*, *GCLM*) and suppress pro-inflammatory mediators (e.g., *NOS2*, *IL-6*, *COX-2*, *NF-kB*, *TNF-α*). AMPK further enhances Nrf2 activation. Reproduced from [35]. Licensed under CC BY 4.0. Abbreviations: Akt, protein kinase B; AMPK, 5′-Adenosine monophosphate-activated protein kinase; ARE, antioxidant response element; COX-2, cyclooxygenase 2; GCLC, glutamate-cysteine ligase catalytic subunit; GCLM, glutamate-cysteine ligase modifier subunit; GPX, glutathione peroxidase; GSK-3β, Glycogen synthase kinase-3β; HO-1, heme oxygenase-1; IL-6, interleukin-6; Keap1, Kelch-like ECH-associated protein 1; NF-kB, nuclear factor kappa beta; NOS2, nitric oxide synthase 2; NQO1, NAD(P)H quinone oxidoreductase 1; Nrf2, nuclear factor erythroid-derived 2-related factor 2; PI3K, phosphoinositide-3 kinase; ROS, reactive oxygen species; sMafs, small musculoaponeurotic fibrosarcoma proteins; TNF-α, tumor necrosis factor-α; Ub, ubiquitin. Arrows indicate relative changes (↑ increase, ↓ decrease, → causative or regulatory relationship, ⊢ inhibitory relationship).

Under basal conditions, Keap1 sequesters Nrf2 in the cytoplasm and promotes its ubiquitination and proteasomal degradation [40]. Keap1 contains multiple cysteine residues that act as redox sensors. Oxidative or electrophilic stress modifies these residues, disrupting the Keap1–Nrf2 complex and allowing Nrf2 stabilization and nuclear translocation [41]. Once in the nucleus, Nrf2 binds ARE sequences and activates transcription of genes encoding antioxidant enzymes and cytoprotective proteins [42] (Figure 2).

Importantly, Nrf2 signaling has been implicated in mitochondrial biogenesis, mitophagy, and regulation of neuroinflammation [43,44]. Given the central contribution of oxidative stress to AD pathophysiology, pharmacological activation of Nrf2 represents an attractive therapeutic strategy [35].

### 1.3. Dimethyl Fumarate: From Traditional Medicine to Neurotherapeutics

Dimethyl fumarate (DMF) is an α,β-unsaturated ester of fumaric acid (C_6_H_8_O_4_; molecular weight 144.13 g/mol) originally derived from the medicinal plant *Fumaria officinalis*, historically used for inflammatory and dermatological conditions [45,46]. The therapeutic properties of *Fumaria officinalis* are attributed to its bioactive alkaloids, flavonoids, and phenolic compounds, which confer antioxidant and anti-inflammatory activity [47,48].

Clinically, DMF has been used for over five decades, initially in psoriasis and later approved in Germany in 1994 for this indication [49]. It is now also approved by the FDA for the treatment of multiple sclerosis, where it exerts immunomodulatory and neuroprotective effects [50].

After oral administration, DMF is rapidly converted by intestinal esterases to its active metabolite, monomethyl fumarate (MMF), and functions as a prodrug [51]. DMF activates the Nrf2 pathway through electrophilic modification of Keap1 cysteine residues, thereby inducing antioxidant and cytoprotective gene expression [52,53]. In addition to redox modulation, DMF influences immune responses by altering lymphocyte subpopulations, inducing apoptosis in activated T cells, and reducing inflammatory signalling [54,55].

Because oxidative stress and neuroinflammation are central to AD progression, and given DMF’s established safety profile in other chronic diseases, repurposing DMF for AD has emerged as a promising strategy [55,56,57]. Preclinical evidence suggests that DMF reduces oxidative damage, modulates inflammatory pathways, and may attenuate Aβ and tau pathology.

### 1.4. Rationale and Objectives of the Present Systematic Review

Despite advances in understanding AD pathogenesis, current therapies remain largely symptomatic and do not effectively halt disease progression [15]. Considering the multifactorial nature of AD and the pivotal role of oxidative stress, pharmacological activation of the Nrf2 pathway represents a rational disease-modifying approach.

This systematic review aims to collect, synthesize, and critically evaluate the available preclinical evidence regarding the therapeutic potential of DMF in AD models. Particular emphasis is placed on:

1. Activation of the Nrf2/ARE signaling pathway,
2. Modulation of oxidative stress and antioxidant responses,
3. Effects on amyloid-β and tau pathology,
4. Anti-inflammatory and neuroprotective mechanisms, and
5. Functional outcomes, including learning and memory performance.

## 2. Methods

### 2.1. Search Strategy

The articles were retrieved from the Scopus and PubMed databases after systematic search from October to December 2025. These databases together provide extensive coverage of biomedical and interdisciplinary research. Due to resource and access limitations, additional databases such as Web of Science and Embase were not included. Grey literature (e.g., conference abstracts, dissertations, and preprints) was not systematically searched. The last search was conducted on 15 December 2025 for PubMed and 17 December 2025 for Scopus. The main keywords used were “Alzheimer’s disease” OR “AD” AND “Dimethyl fumarate” OR “DMF” AND “Nrf2” AND “Neuroinflammation” AND “Ferroptosis”. The full search strategy is presented in Supplementary Table S1. This systematic review was conducted in accordance with the PRISMA 2020 guidelines, and it was not registered, hence no review protocol was created for it.

### 2.2. Study Inclusion and Exclusion Criteria

The literature eligible for inclusion in this systematic review consisted of primary preclinical or clinical research articles presenting original results, which were identified using the respective filter in each database. No time restriction was imposed on the publication date of the studies and the literature search included articles published up to December 2025.

The exclusion criteria were as follows:

- Review articles or meta-analyses.
- Non-English articles.
- Articles that were not primary research reports.
- Studies that did not specifically address the selected keywords.
- Studies not related to the treatment of Alzheimer’s disease specifically.
- Studies in which DMF was not used as a therapeutic agent.
- Articles for which full-text access was not available.

### 2.3. Study Quality Assessment

The methodological quality of the included studies was carefully assessed according to the specific methodology used in each study. Studies were grouped according to their methodological approach into in vitro, in vivo, and mixed-methodology categories. Risk of bias in the in vivo preclinical studies and the in vivo part of the mixed-methodology studies was evaluated using a structured assessment based on the SYRCLE Risk of Bias (RoB) tool, an adaptation of the Cochrane Risk of Bias tool specifically for animal intervention studies [58]. For each study, predefined questions directly reproduced from the tool (Table 1) were systematically applied, and the answers were assigned accordingly to determine the overall risk of bias.

The methodological quality of the in vitro studies and the in vitro part of the mixed-methodology studies was assessed using an adapted checklist based on the Quality Assessment Tool For In Vitro Studies (QUIN) tool [59] and Science in Risk Assessment and Policy (SciRAP) framework [60], including items related to randomization, blinding, replication, standardized culture conditions, outcome measurement, and statistical reporting. The QUIN tool and the SciRAP framework are structured evaluation approaches designed to improve transparency, reproducibility, and reliability in in vitro research by assessing key methodological domains such as study design, reporting quality, and risk of bias. For each study, predefined questions (Table 2) were systematically applied, and the answers were assigned accordingly to determine the overall risk of bias.

**Figure 3:**
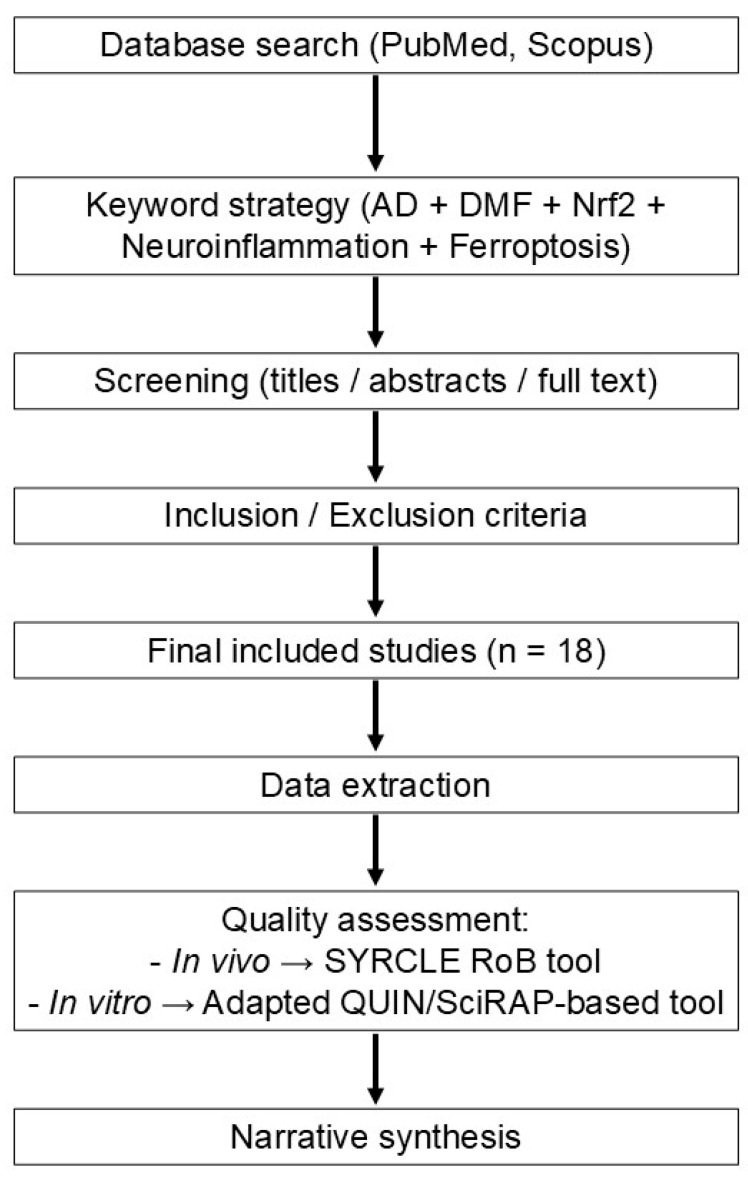
Overview of the methodology of the systematic review.

To improve clarity, a schematic overview of the methodological workflow of this systematic review is presented in Figure 3.

## 3. Results

### 3.1. Selection of Studies

A total of 45 studies were identified from the two selected databases during the search. Two independent reviewers screened titles, abstracts, and full texts. Disagreements were resolved through discussion. Duplicate records were identified and removed after screening the titles and abstracts. The search did not yield any clinical trials that met the inclusion criteria. Therefore, this systematic review focuses exclusively on preclinical studies.

After the removal of 15 duplicate studies, 30 articles remained and were further assessed for eligibility based on the predefined inclusion and exclusion criteria. Following the full-text assessment of the 29 remaining studies, as 1 was not retrieved, 11 were excluded because they did not meet the inclusion criteria (Supplementary Table S2). The remaining studies fulfilled all eligibility requirements and were included in the systematic review. Therefore, in total, 18 preclinical studies investigating DMF in in vitro and in vivo models of AD were identified and analysed. Data extraction was performed independently by two reviewers. The following data were extracted: study design, experimental model, DMF dose, outcome measures (oxidative stress markers, inflammatory markers, amyloid/tau pathology, cognitive outcomes), and key findings. Given the limited availability of clinical data, the present review focuses primarily on mechanistic and experimental evidence to evaluate whether DMF demonstrates sufficient translational potential to justify further clinical investigation.

**Figure 4:**
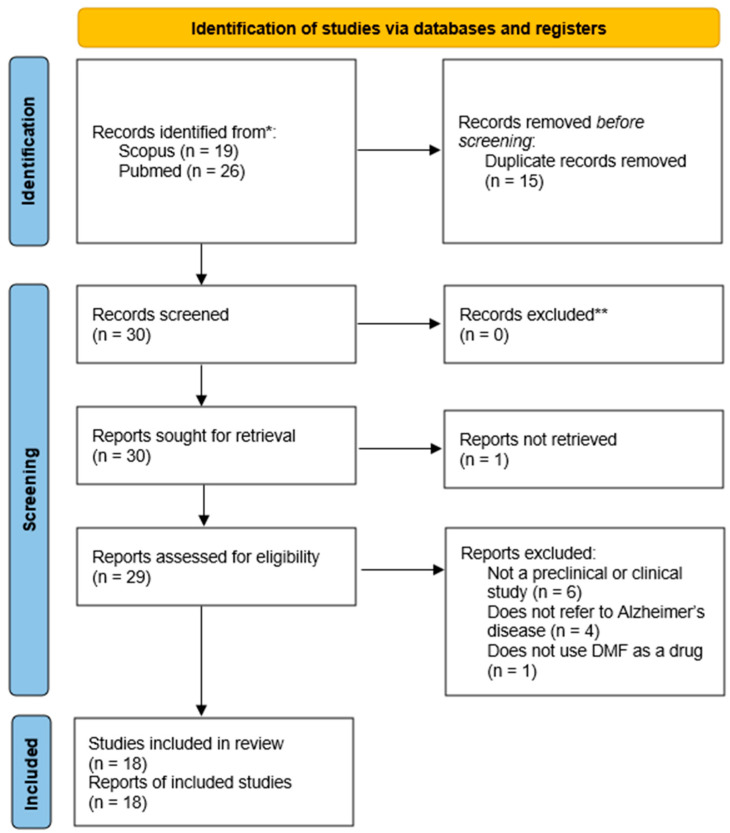
PRISMA flow chart for the study selection process. * The number of records identified from each database searched was reported. ** No automation tools were used to exclude studies. Adapted from [61].

Figure 4 presents the PRISMA flow chart illustrating the study selection process, along with the number of articles screened, excluded, and ultimately included in the review.

A quantitative meta-analysis was not performed due to the considerable heterogeneity among the studies included. The studies varied widely in terms of experimental models, including different in vitro systems and animal models, as well as in DMF dosing, treatment duration, and the types of outcomes assessed, such as biochemical, histological, and behavioural measures. In addition, the relatively small number of studies within each subgroup made meaningful statistical pooling impractical. For these reasons, a narrative synthesis was considered the most appropriate approach to summarise and interpret the available evidence. Additional well-designed preclinical and clinical studies are definitely needed to meet established methodological quality criteria and to generate more robust and less biased evidence.

### 3.2. Study Quality

**Figure 5:**
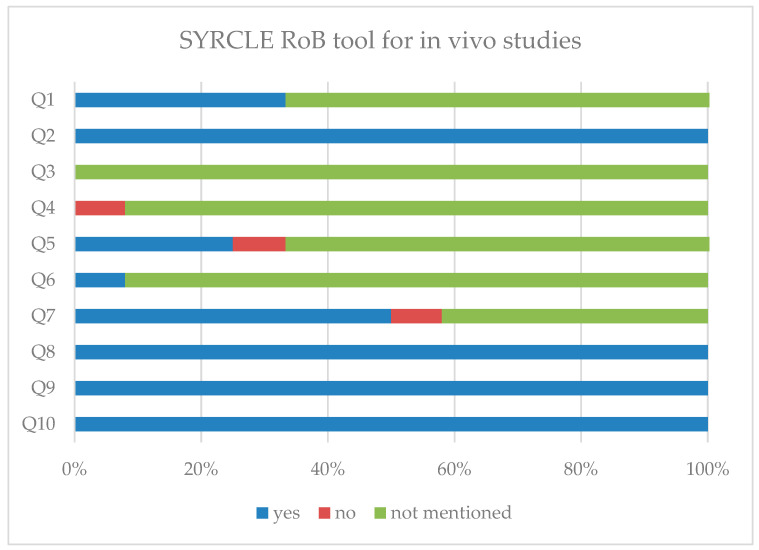
Risk of bias assessment of the included in vivo preclinical studies. Risk of bias was evaluated using the SYRCLE RoB tool. The figure summarizes the responses for each RoB question across the included in vivo studies, illustrating the distribution of low, high, or unclear risk of bias judgments.

**Figure 6:**
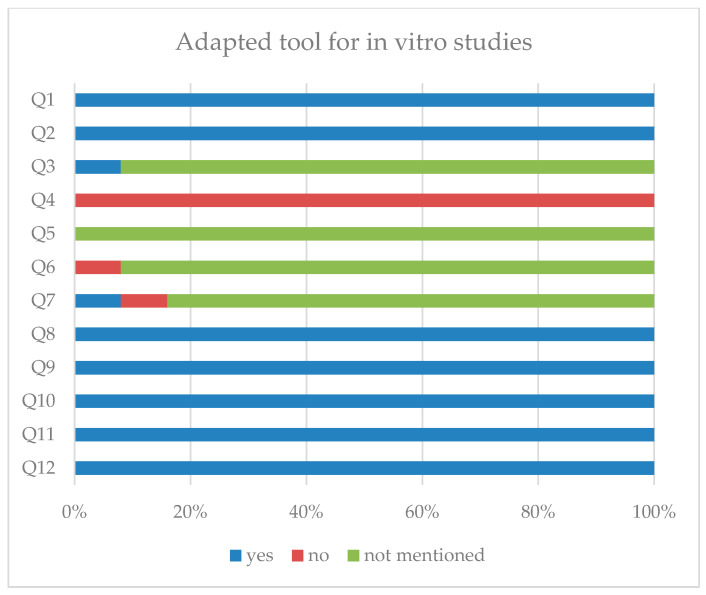
Methodological quality assessment of the included in vitro studies. The methodological quality and risk of bias of the in vitro studies were evaluated using an adapted checklist based on the QUIN and SciRAP frameworks. The figure summarizes the distribution of responses for each assessment item across the included studies.

The methodological quality of the included studies is presented in Figure 5 and Figure 6. Quality assessment was performed independently by two reviewers. Detailed risk of bias assessments per study are provided in Supplementary Tables S3 and S4. Despite the fact that in vitro studies are generally less clinically translatable, as they are conducted on isolated cells rather than whole organisms, all retrieved studies were incorporated into our analysis for a more comprehensive preclinical overview. Overall, the included studies were considered to be of adequate methodological quality. As illustrated in the figures, most domains were rated as having a low or acceptable risk of bias, suggesting that the majority of studies followed basic methodological standards. However, several domains were classified as unclear, primarily due to incomplete reporting rather than clear evidence of bias, and a small number of studies showed a higher risk of bias, particularly in areas such as randomization, blinding, and outcome assessment. These methodological limitations should be taken into account when interpreting the reported beneficial effects of DMF, as they may contribute to an overestimation of treatment effects. Nevertheless, the main sources of bias commonly associated with preclinical research appeared to be generally addressed across the included studies.

### 3.3. Analysis of Included Studies

Studies were grouped according to their methodological approach into in vitro, in vivo, and mixed-methodology categories to facilitate a more coherent and meaningful interpretation of the findings. Given the variability in experimental conditions and the limited number of studies within each subgroup, no formal subgroup or sensitivity or overall certainty of the evidence (e.g., GRADE) analyses were performed. Instead, the synthesis focused on identifying patterns and mechanistic insights across studies, particularly in relation to oxidative stress modulation, neuroinflammatory processes, and Nrf2 pathway activation in AD.

#### 3.3.1. In Vitro Studies

Six of the 18 preclinical studies included in the results consisted exclusively of in vitro experiments and one also included ex vivo experiments. These studies are presented below.

Rajput et al. [62] studied the effect of DMF on the phosphorylation of tau protein, formation of Aβ fibrils, and microtubule degradation in SH-SY5Y neuroblastoma cells. A change in cell apoptosis and inhibition of cytochrome c (Cyt c) expression by DMF was observed. DMF also reduced tau phosphorylation at Ser396 and Thr23 sites via the glycogen synthase kinase 3 beta (GSK-3β) pathway. However, it did not cause significant changes in p38 and c-Jun N-terminal kinase (JNK), which belong to the group of mitogen-activated protein kinases (MAPKs) associated with microtubules and leading to tau protein phosphorylation, compared to cells treated with Aβ_1–42_. DMF also preserved the expression of β-catenin and cyclin D1 by acting on the Wnt/β-catenin and the phosphatidylinositol 3-kinase/protein kinase B (PI3K/AKT) pathway. Finally, it prevented the degradation of microtubules in cells treated with Aβ_1–42_ because it protected β-tubulin and α-tubulin, and even prevented the formation of Aβ fibrils and prevented the cytotoxicity caused by Aβ_1–42_.

In another study by the same group [63], it was examined whether DMF affects the activity of protein phosphatase 2B (PP2B) (or calcineurin) and its downstream targets in SH-SY5Y neuroblastoma cell line exposed to Aβ_1–42_. DMF reduced the cell death marker lactate dehydrogenase (LDH) and decreased cell mortality in a dose-dependent manner. It also reduced the levels of PP2B. No change was observed in other protein phosphatases such as phosphatase type 1 (PP1) and phosphatase type 2A (PP2A). DMF also reduced BAD dephosphorylation mediated by PP2B activation in a dose-dependent manner, as well as preventing cAMP response element-binding protein (CREB) transcription factor phosphorylation by Aβ_1–42_. Furthermore, DMF reduced the expression of the nuclear factor of activated T cells 1 (NFAT1) in cells exposed to Aβ_1–42_, and since NFAT1 expression induces β-secretase (BACE1) protein expression, DMF also reduced BACE1 expression by reducing nuclear factor-kappa B (NF-kB)-mediated *BACE1* gene expression, thus exhibiting neuroprotection. Finally, DMF had no effect on the amyloid precursor protein (APP), which regulates cell growth and survival.

In addition, the study by Campolo et al. [64] evaluated the effects of DMF in SH-SY5Y cells in vitro and in an ex vivo model of organotypic hippocampal slices. In vitro, DMF improved cell viability in SH-SY5Y cells stimulated with Aβ_1–42_ and reduced tau expression and phosphorylation. It also increased Nrf2 and Keap1 protein levels, as well as heme oxygenase-1 gene (*Hmox1*) and manganese superoxide dismutase gene (*SOD2*) expression. DMF also elevated reduced glutathione (GSH) levels, decreased malondialdehyde (MDA), a product of lipid peroxidation, and lowered intracellular ROS concentration. In addition, it reduced NF-kB nuclear translocation and maintained the activity of IkB-a in the cytoplasm, since it is degraded under inflammatory conditions. In the absence of Nrf2, DMF lost its protective effect. In the ex vivo hippocampal slice model, DMF protected cell viability and reduced the expression and phosphorylation of tau protein.

In the study by Silva et al. [65], DMF was tested in mouse neuronal cell lines (N2a), either wild type (N2a-wt) or overexpressing the human APP (N2a-APPwt), which served as models of AD. In addition, mouse microglial BV-2 cells stimulated with lipopolysaccharide (LPS) were used as a model of inflammation. The results showed that DMF activated Nrf2 in N2a-APPwt cells and increased the expression of *Hmox1*, which depends on Nrf2 activation and plays a key role in antioxidant and anti-inflammatory mechanisms. Heme oxygenase-1 (HO-1) protein levels were also increased in these cells. Furthermore, DMF maintained activation of protein kinase B (PKB), an upstream activator of Nrf2. Since intracellular calcium signalling is important for cellular homeostasis, calcium levels were also assessed. However, DMF did not reverse the altered mitochondrial or intracellular calcium levels. In BV-2 cells, DMF significantly decreased the expression of *iNOS* and *IL-1β* genes and caused a small, non-significant reduction in tumour necrosis factor-α (TNF-α) mRNA. It also reduced inducible nitric oxide synthase (iNOS) protein levels, reversed the increase in pro-interleukin-1β (pro-IL-1β), and decreased IL-1β secretion in microglial cells.

Another in vitro study recorded the effects of DMF on the regulation of metabolic stress and neuroinflammation in neurons caused by exposure to low and high concentrations of sucrose and glucose in the SH-SYS5Y cell line. In two groups of cells, one without glucose (GD) and the other with high glucose concentration (HG), DMF increased viability by causing dose-dependent protection. In terms of oxidative stress regulation, the GD group had increased expression of HO-1, which was reduced by DMF, and had decreased expression of the enzyme manganese superoxide dismutase (MnSOD), while after DMF its concentration quadrupled. Furthermore, GD reduced catalase and Nrf2 levels, while DMF prevented this reduction in a dose-dependent manner. GD also reduced GSH and increased the reactive oxygen species modulator 1 (ROMO1) protein, which DMF reversed. DMF also reduced neuroinflammation through the NF-kB pathway. Specifically, the negative regulators of NF-kB, IkB-a and optineurin (OPTN), were examined, the levels of which were reduced in GD, and DMF counteracted this change. In contrast, in the HG group, the expression of HO-1 and MnSOD was reduced, which was increased by DMF. Furthermore, in HG, GSH and ROMO1 levels were reduced, but treatment with DMF at the highest concentration (1mM) worsened GSH levels caused by high sucrose concentration and increased ROMO1 levels, while at a concentration of 0.1 mM it stopped the decrease in GSH. In addition, in HG, NF-kB expression was increased due to high sucrose, and DMF prevented this increase. In both groups, p53 expression was reduced, and DMF restored the reduction in a dose-dependent manner, thus enhancing the nervous defence system. Finally, Nrf2 expression was reduced in both groups to study the effect of DMF. In both groups, DMF did not inhibit glucose-induced cell death. Due to the reduced expression of Nrf2, HO-1 and MnSOD levels were reduced and DMF was unable to increase them [66].

Another experimental study examined whether DMF, through activation of the Nrf2 pathway, can stop the accumulation of proteins modified with hydroimidazolone 1 (MG-H1), which originate from the highly reactive carbonyl compound methylglyoxal (MGO). The results of DMF demonstrated the accumulation of Nrf2 in the nucleus and an increase in GSH concentration in SH-SY5Y cells. DMF also increased the expression of the enzyme glutamate-cysteine ligase modifier subunit (GCLM), which increases the rate of GSH synthesis. DMF thus stopped the accumulation of MG-H1 by stimulating GSH production and activating the Nrf2 pathway [67].

#### 3.3.2. Mixed-Methodology Studies

Five of the 18 analysed studies included both in vitro and in vivo results and are presented below.

In vitro experiments using the T98G astrocyte cell line showed that DMF induced nuclear translocation of Nrf2, indicating activation of the Nrf2 pathway. In A1 astrocytes, DMF significantly increased the expression of Nrf2 and its target genes, while inhibiting the expression of *H2d*, *H2t23*, *Gbp2*, *C3*, and *Socs3*. However, in A1 astrocytes with insufficient Nrf2, DMF did not affect the expression of these genes. DMF also reduced the levels of complement protein C3 and suppressor of cytokine signalling 3 (SOCS3) in A1 astrocytes. In the subsequent in vivo experiments using App^NL-G-F/NL-G-F^ (App-KI) and WT mice, DMF administration increased the expression of *GCLM* in microglial cells. In astrocytes, the expression of *NQO1* and *Osgin1* increased, while no difference in *NFe2L2* expression was observed between microglia and astrocytes. In addition, DMF improved cognitive function in App-KI mice. It also reduced neuroinflammation by inhibiting reactive astrocyte markers and blocking the signalling pathways of signal transducer and activator of transcription 3 (STAT3) and SOCS3. Finally, DMF reduced dystrophic neurites without affecting Aβ clearance in App-KI mice [68].

Another combined in vitro and in vivo experimental study by Sun et al. [57] investigated whether DMF treatment could slow the progression of Alzheimer’s disease through its antioxidant effects and activation of the Nrf2 pathway. In vitro experiments in hippocampal neurons from embryonic mice showed that DMF exhibited antioxidant activity and increased neuron survival under oxidative stress conditions. In neurons where the Nrf2 pathway was suppressed, ROS levels increased under stress conditions, whereas in neurons with active Nrf2, ROS levels remained stable, indicating the protective role of DMF through the Nrf2 pathway. In the in vivo part of the study, five groups were created in mouse models of Alzheimer’s disease (WT, AD, AD + DMF, Nrf2-KO + AD, and Nrf2-KO + AD + DMF). In an escape latency experiment, the AD + DMF group showed the shortest escape time, suggesting that DMF improved cognitive impairment through activation of the Nrf2 pathway. In this group, DMF also delayed Aβ-induced hippocampal atrophy and increased antioxidant enzyme levels through Nrf2 activation. In addition, DMF treatment suppressed lipid peroxidation, apoptosis, mitochondrial dysfunction, and inflammation, and reduced Aβ deposition.

Babaei et al. [69] investigated the effect of DMF on mesenchymal stem cell (MSC) therapy in a rat model of AD using both in vitro and in vivo experiments. The in vitro results showed increased proliferation and survival of MSCs treated with DMF, along with a significant increase in Nrf2 expression, suggesting the antioxidant capacity of DMF. In the in vivo experiments, three groups were studied (AD, AD + MSC, and AD + MSC + DMF). In an escape latency test, the AD + MSC + DMF group showed the shortest escape time, indicating that co-administration of DMF enhanced the effectiveness of MSC therapy. This group also showed increased mRNA levels of brain-derived neurotrophic factor (BDNF) and nerve growth factor (NGF) compared with the other groups. In addition, regarding apoptotic markers, the AD + MSC + DMF group showed increased expression of the *Bcl2* gene and decreased expression of *Bax*, *Caspase 3*, and *Cyt c* genes.

In another experimental study, the potential effects of DMF on microglia (MG) activated during systemic inflammation were investigated, as such activation can lead to neuroinflammatory responses. In vitro experiments using LPS-treated microglia showed that DMF suppressed microglial activation by inhibiting the expression of their maturation markers. It also increased the expression of Nrf2 target genes and inhibited the mRNA expression of inflammatory cytokines TNF-α, IL-1β, and iNOS, as well as interleukins IL-23p19 and IL-12p40, which were induced in microglia by LPS. Furthermore, DMF inhibited NF-kB activation and reduced microglia-induced neurotoxicity and both acute and chronic neuroinflammation. In the in vivo experiments, DMF treatment alleviated cognitive impairments in LPS-treated mice [70].

Huang et al. [71] conducted both in vivo and in vitro experiments using transgenic APP/PS1 mice and HT22 mouse hippocampal cell lines, respectively, to evaluate the therapeutic effects of the compound HY-021068 (HY) in combination with DMF. The in vivo results showed that the HY + DMF treatment improved learning and memory deficits in APP/PS1 mice. The combination also reduced Aβ accumulation and Aβ_1–42_ production in the hippocampus and cerebral cortex and decreased tau protein phosphorylation. In addition, it reduced ROS levels in brain tissue and activated the Nrf2 pathway by increasing the expression of the antioxidant enzymes HO-1 and NAD(P)H quinone oxidoreductase 1 (NQO1). The treatment also increased GSH levels and glutathione peroxidase 4 (GPX4) expression while reducing iron loss in APP/PS1 mice. In the in vitro experiments, the results showed that in HT22 cells where Nrf2 was not activated by DMF, HY was unable to exert its anti-ferroptotic and antioxidant effects.

#### 3.3.3. In Vivo Studies

The remaining seven studies involved experiments conducted in in vivo models and are described below.

In the study by Majkutewicz et al. [72], the authors examined whether DMF could prevent spatial memory weakening and hippocampal neurodegeneration in rodent models of AD. DMF treatment reduced memory impairment and had a significant positive effect on spatial memory acquisition. It also reduced neurodegeneration in the hippocampus, particularly in the CA1 and CA3 regions, and increased neuron density in the CA1 region, demonstrating the neuroprotective properties of DMF. In addition, DMF limited neuroinflammatory responses by regulating the expression of IL-6 and IL-10 in the hippocampus. However, it did not prevent neurodegeneration in the dentate gyrus (DG) of the hippocampus.

In a subsequent study, the same researchers investigated how age influences the effects of DMF treatment in young and aged rats with AD. In swimming, detection, distance, and memory tests, DMF treatment was more effective in older rats, as they showed better spatial memory performance. Older rats also had a higher number of microglial cells than young rats, with increased activation of the microglial marker CD68. Treatment with DMF reduced CD68 expression in the DG of the hippocampus, indicating an effect on microglial activation. DMF also influenced the expression of the cytokine IL-10 in the CA3 region of the hippocampus, where the number of IL-10-positive cells was higher in young rats than in aged rats. The oxidative stress marker nitrotyrosine 3 (NT3) was elevated in all hippocampal regions of older rats and in the CA1 and DG regions of young rats. Overall, DMF prevented neurodegeneration in both age groups [73].

The potential effect of DMF on dementia associated with postmenopause was also examined in an ovariectomized and D-galactose-treated (OVX/D-Gal) rat model of AD. DMF significantly improved general activity and cognitive function in OVX/D-Gal rats, particularly by improving spatial working memory and learning ability. It also increased the number of intact neurons in the CA1 region of the brain, thereby reducing the level of neurodegeneration. DMF treatment reduced the immunoreactivity of glial fibrillary acidic protein (GFAP) in the hippocampus, as the OVX/D-Gal rats showed severe astrogliosis that was alleviated after DMF administration. In addition, DMF activated the adenosine monophosphate-activated protein kinase/sirtuin 1 (AMPK/SIRT-1) and AKT/CREB/BDNF pathways in the hippocampus, which are involved in neuronal formation and survival. OVX/D-Gal rats showed increased levels of hippocampal adiponectin and its type I receptor, and these levels were reduced after DMF treatment. DMF also inhibited neuroinflammation and oxidative stress caused by NF-kB activation in OVX/D-Gal rats and helped restore redox balance. Finally, DMF reduced the levels of BACE1 and Aβ_42_ markers in the hippocampus and decreased the expression of p-GSK-3β and p-tau proteins [74].

Rojo et al. [75] evaluated the role of Nrf2 in the inflammatory response in a mouse model combining amyloidosis and tauopathy. This model exhibits motor and cognitive impairments similar to those observed in AD and involves neuronal expression of the hAPPV717I and hTAUP301L proteins, which correspond to amyloidosis and tauopathy. The study also examined the potential use of DMF as an anti-inflammatory treatment. Experiments conducted in Nrf2 knockout and wild-type mice showed that the absence of Nrf2 worsened the inflammatory response, leading to an earlier onset and greater severity of symptoms. When Nrf2 activation by DMF was evaluated, the results showed a significant increase in Nrf2 expression in the brain. DMF also improved GFAP levels in astrocytes in the hippocampus and brainstem, indicating preservation of neural tissue. In addition, DMF reduced microgliosis and showed a decreasing trend in the levels of pro-inflammatory mediators cyclooxygenase-2 (COX-2) and NADPH oxidase 2 (NOS-2), as well as the glial markers GFAP, ionized calcium-binding adapter molecule 1 (IBA1), and major histocompatibility complex II (MHCII). Finally, DMF slowed the progression of motor impairments and improved memory function. Although the differences were modest, the trend was statistically significant after two weeks, suggesting that DMF can alleviate brain inflammation and the motor and cognitive impairments caused by the expression of the *hAPPV717I* and *hTAUP301L* genes.

Wrona et al. [76] used two groups of mice, young (4 months old) and aged (22 months old), and administered streptozotocin (STZ) via the intracerebroventricular route (ICV) to induce AD-like neuroinflammation. The aim was to examine the immunological differences between aging and AD and to evaluate the effect of DMF. In detection and spatial memory tests, aged mice showed greater impairment in memory acquisition, which was reduced after DMF treatment. DMF reduced peripheral neuroinflammation and induced an anti-inflammatory response in aged mice, but not in young mice. Specifically, after DMF treatment in aged mice, the total number of B lymphocytes, natural killer cells, and T CD3^+^CD4^+^CD8^−^ and T CD3^+^CD4^−^CD8^+^ lymphocytes decreased, while the number of T CD3^+^ peripheral lymphocytes and peripheral monocytes increased. In addition, DMF limited the production of pro-inflammatory cytokines such as interferon-γ (IFN-γ) and IL-6 and enhanced the ability of peripheral blood mononuclear cells (PBMCs) and smooth muscle cells (SMCs) to produce the anti-inflammatory cytokine IL-10. Finally, in aged mice, DMF treatment led to reductions in the number of red blood cells, haematocrit, haemoglobin concentration, red blood cell distribution width, and mean platelet volume.

To investigate the synergistic effect of DMF with vitamin D3 (VITD3), another study examined rats treated with STZ. Treatment with vitamin D3 and DMF was evaluated at three time points: before stereotactic surgery (T1), 45 days after the onset of Alzheimer’s symptoms (T2), and at cardiac puncture on day 90 (T3). The combination group (STZ + VITD3 + DMF) showed increased levels of the vitamin D metabolite 25(OH)D3 in blood plasma at T1 and T2, with no change at T3. It also showed a decrease in the 24,25(OH)_2_D_3_/25(OH)D_3_ ratio at T2 and T3, along with an increase in the 3-epi-25(OH)D_3_/25(OH)D_3_ ratio. The combination group demonstrated gradual improvement in spatial memory from T1 to T3. In addition, there was a greater reduction in tau protein levels in the CA1–CA3 regions of the hippocampus and in the DG compared with the group treated with DMF and VITD3 separately. Rats in the combination group also showed reduced lipid peroxidation and increased GSH levels, a change that was also observed in the DMF group. Finally, the DMF, VITD3, and combination groups showed decreased levels of TNF-α and elevated levels of the anti-inflammatory cytokine IL-10, while the DMF and combination groups also showed a moderate reduction in IL-6 [77].

Finally, the study by Möhle et al. [78] investigated the effect of DMF in female mouse models of AD characterized by β-amyloidosis. However, unlike previous studies, the researchers found that treatment with DMF did not significantly affect learning or memory performance compared with placebo. In addition, in two groups of mice aged 80 and 100 days, DMF did not alter the accumulation of Aβ protein in the brain. Finally, DMF treatment did not produce significant differences in immune system markers or in microglial activation between the two groups of mice.

Table 3 below presents the main characteristics of the studies that were analysed, including the exact models used, the doses of DMF and other applied interventions, and the main results derived from each study.

## 4. Discussion

This systematic review brought together evidence from 18 preclinical studies exploring the potential therapeutic effects of DMF in experimental models of AD. Overall, the findings suggest that DMF exerts a variety of neuroprotective effects, mainly through activation of the Nrf2 signalling pathway and the subsequent enhancement of cellular antioxidant responses. Across in vitro, in vivo, and mixed-methodology studies, DMF treatment was generally associated with reduced oxidative stress, decreased inflammatory activity, and improved behavioural outcomes in several rodent models. However, the strength of this evidence varies depending on the mechanism examined, and there is considerable heterogeneity in the experimental models, treatment approaches, and outcome measures used. This variability makes it more difficult to draw firm conclusions about the overall therapeutic potential of DMF and highlights important limitations when considering its relevance to clinical settings. Importantly, the interpretation of the findings should be considered in light of the methodological quality of the included studies. Some studies were associated with unclear or high risk of bias, particularly due to insufficient reporting of randomization, blinding, and sample size justification. These factors may increase the likelihood of overestimating treatment effects. Consequently, the overall conclusions of this review should be interpreted with caution, and the apparent consistency of positive findings should not be taken as definitive evidence of efficacy. Future studies employing rigorous experimental design and standardized reporting are required to validate these findings.

One of the most consistent findings across the included studies was the activation of the Nrf2 pathway and the resulting enhancement of antioxidant defence mechanisms. Several in vitro studies showed increased nuclear accumulation of Nrf2 alongside upregulation of downstream antioxidant enzymes such as HO-1, MnSOD, and NQO1 following DMF treatment. In neuronal cell models, including SH-SY5Y and N2a cells, DMF was also found to increase GSH levels and reduce markers of oxidative stress. Similar patterns were observed in animal studies, where DMF administration led to increased antioxidant enzyme expression and reduced lipid peroxidation in brain tissue. Taken together, these consistent findings across both cellular and animal models support the idea that Nrf2 activation represents a key and reliable mechanism underlying the neuroprotective effects of DMF in AD-related contexts.

In addition to its antioxidant properties, DMF also appears to have notable anti-inflammatory effects. A number of studies reported that DMF reduces the expression of pro-inflammatory mediators and limits microglial activation. For instance, decreases in cytokines such as TNF-α, IL-1β, and iNOS were observed in stimulated microglial cells, along with inhibition of the NF-kB signalling pathway. These findings were supported by in vivo studies, which showed reductions in neuroinflammatory markers such as COX-2, NOS-2, GFAP, and IBA1 following DMF treatment. Importantly, these anti-inflammatory effects were often accompanied by improvements in cognitive performance in behavioural tests. Together, this suggests that DMF may help mitigate neuroinflammation, a central process contributing to neuronal dysfunction and disease progression in AD.

**Figure 7:**
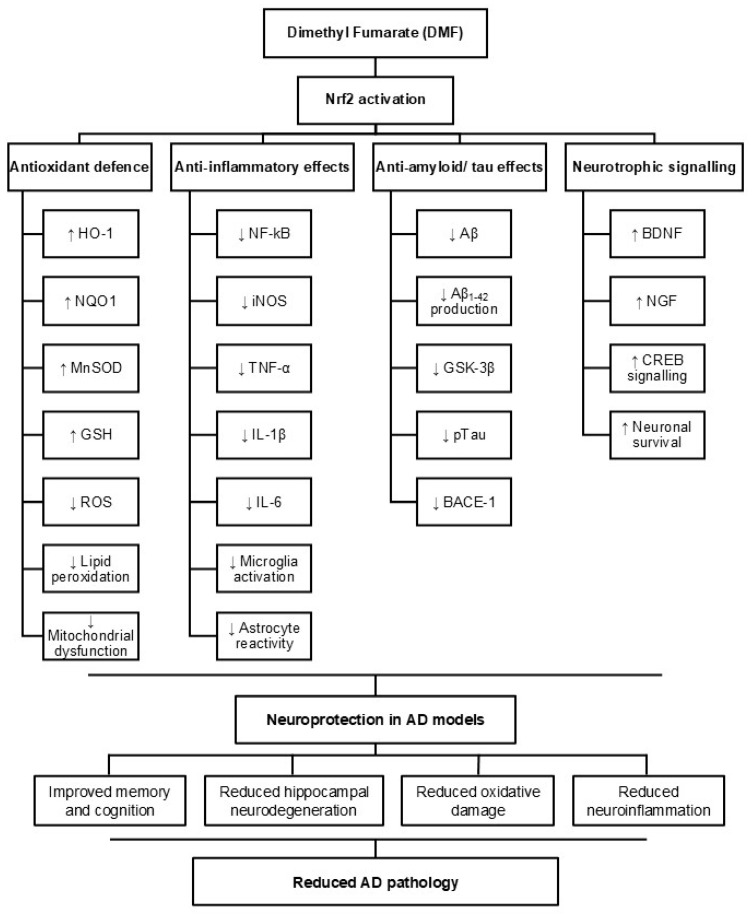
Proposed mechanisms of DMF in Alzheimer’s disease. DMF activates the Nrf2 signalling pathway, leading to increased expression of antioxidant enzymes and enhanced glutathione synthesis. This activation reduces oxidative stress and suppresses NF-kB-mediated inflammatory signalling. Through these mechanisms, DMF decreases Aβ accumulation, tau phosphorylation, and neuroinflammation while promoting neuronal survival pathways. Collectively, these effects may contribute to reduced neurodegeneration and improved cognitive function in preclinical models of Alzheimer’s disease. Arrows indicate relative changes (↑ increase, ↓ decrease).

While antioxidant and anti-inflammatory effects were consistently reported, the evidence for direct effects of DMF on the core pathological hallmarks of AD, including Aβ accumulation and tau phosphorylation, was less consistent. Some studies did report reductions in Aβ production, tau phosphorylation, or related signalling pathways. For example, DMF was shown to reduce tau phosphorylation and protect against microtubule degradation in neuronal models exposed to Aβ toxicity [62]. In certain animal studies, reductions in Aβ deposition and phosphorylated tau were also observed [57,71,74,77]. However, these findings were not uniform across all studies. Notably, one study using APPPS1–21 transgenic mice found no significant effects of DMF on memory performance, amyloid accumulation, or immune markers [78]. This variability suggests that, although DMF may influence amyloid and tau pathology under certain conditions, these effects are less consistent than its antioxidant and anti-inflammatory actions. Figure 7 summarizes the discussed mechanisms of DMF in AD.

The diversity of experimental models used across studies likely contributes to the observed variability. The included studies employed a wide range of models, including transgenic amyloid models, metabolic models induced by ICV-STZ, inflammatory models using LPS, and hormone- or metabolism-based models such as ovariectomy combined with D-gal treatment. Each of these models reflects different aspects of AD pathology and may therefore respond differently to treatment. For example, STZ models primarily capture metabolic dysfunction and neuroinflammation, whereas transgenic APP models focus more specifically on amyloid accumulation [79]. As a result, findings observed in one model may not necessarily translate to others, making direct comparisons more challenging and emphasizing the need to evaluate DMF across multiple systems.

Another important limitation is the variation in dosing regimens and treatment protocols. In vitro studies used a wide range of concentrations, with 30 μM being the most common one, while in vivo studies administered doses spanning from approximately 45 to 300 mg/kg. In preclinical in vivo studies, DMF is most commonly administered at doses ranging from approximately 25 to 100 mg/kg, with 50–100 mg/kg representing the most frequently used and biologically effective range. Higher doses are often reported as maximally effective, while doses above 160 mg/kg may be associated with toxicity [80], although this was not apparent in the study of Wang et al. [68] who used 300 mg/kg of DMF for their experiments. While these doses are generally considered to approximate the exposure achieved with the clinically approved dose of 240 mg twice daily used in multiple sclerosis patients, direct translation between animal and human dosing remains complex. Using standard body surface area normalization, the corresponding human equivalent doses are substantially lower, and interspecies differences in metabolism, bioavailability, and administration routes must be taken into account. Following oral administration in humans, DMF is rapidly hydrolysed to its active metabolite MMF, which reaches peak plasma concentrations within approximately 2–2.5 h and is capable of crossing the blood–brain barrier, supporting its central nervous system activity [81,82]. Importantly, the neuroprotective effects observed in preclinical models, including activation of the Nrf2 pathway and modulation of oxidative stress and inflammatory responses, are consistent with mechanisms observed at clinically relevant exposure levels [83]. However, differences in pharmacokinetics and tissue distribution between species highlight the need for cautious interpretation of dose–response relationships and stress out the importance of further studies to better define the translational relevance of DMF dosing in AD. Future studies should also report brain concentrations where possible.

In addition, the timing of treatment varied considerably, with some studies applying DMF before the onset of pathology and others after cognitive deficits had already developed. These differences are likely to influence outcomes, as preventive interventions may show stronger effects than treatments introduced at later stages [84]. The lack of standardized dosing and treatment strategies therefore represents a key limitation in the current evidence base.

Interestingly, some studies indicated that the effects of DMF may depend on the age of the experimental animals. In particular, studies using older rodents reported more pronounced improvements in cognition and greater reductions in inflammatory markers compared to younger animals. This may be explained by the higher baseline levels of oxidative stress and inflammation typically seen in aged animals [85], which could make them more responsive to interventions targeting these pathways. While this raises the possibility that DMF may be especially beneficial in later stages of disease, further research is needed to determine whether similar age-related effects occur in humans.

Another emerging observation is the potential benefit of combining DMF with other therapeutic agents. Two studies reported enhanced effects when DMF was used alongside additional compounds. For example, combining DMF with vitamin D3 resulted in greater improvements in spatial memory, reduced lipid peroxidation, and lower tau levels compared to either treatment alone [77]. Similarly, DMF was shown to enhance the antioxidant and anti-ferroptotic effects of another compound in a transgenic mouse model [71]. These findings support the idea that DMF may be particularly useful as part of a multi-target treatment approach, which is increasingly considered necessary for complex diseases like AD.

Despite these promising findings, several challenges remain when considering translation to clinical practice. Most of the available evidence comes from animal models and immortalized cell lines, which cannot fully capture the complexity of human AD. The disease develops over many years and involves interactions between genetic, metabolic, vascular, and environmental factors, whereas experimental models typically reflect only selected aspects of the pathology. As a result, treatments that appear effective in these simplified systems may not necessarily show the same benefits in patients. In addition, relatively few studies have examined the long-term effects of DMF on disease progression or functional recovery. Having discussed these, it is worth mentioning that a clinical trial scheduled to begin in Poland in 2027 (NCT06850597) aims to assess the safety and efficacy of DMF in patients with mild cognitive impairment and AD-related dementia. This randomized, double-blind, placebo-controlled study will evaluate potential improvements in cognitive functions including memory, language, attention, and executive function. The results of this trial will be essential in determining whether the promising findings from preclinical research can indeed be translated into clinical benefit.

Another limitation is the relatively small number of studies specifically investigating DMF in the context of AD. While DMF has been widely studied in other neurological conditions, particularly multiple sclerosis, its role in AD remains less well explored. Notably, several preclinical studies have shown that DMF can inhibit ferroptosis via Nrf2-related pathways in models of myocardial ischemia and lung injury [55,86]. In more detail, in the study by Li et al. (2025) [86], they observed that DMF, through activation of the Nrf2 pathway in combination with electroacupuncture, exhibited anti-ferroptotic activity in the treatment of myocardial ischemia. In another in vivo study [55] conducted in rats under hypoxic conditions associated with acute lung injury, DMF, by regulating the Nrf2/SLC7A11 pathway, prevented ferroptosis and reduced the inflammatory response in lung epithelial cells. These findings are particularly relevant given that ferroptosis is closely linked to oxidative stress, lipid peroxidation, and iron dysregulation, all of which are key features of AD pathophysiology [87,88,89]. Through activation of Nrf2 and downstream targets such as SLC7A11 and glutathione-related pathways, DMF may therefore affect key mechanisms involved in neuronal vulnerability and degeneration. However, studies specifically examining DMF-mediated ferroptosis inhibition in AD are extremely limited. To date, and based on our literature search, only Huang et al. [71] have investigated this particular mechanism in an AD context, highlighting a significant gap in the literature. Given the established roles of oxidative stress and ferroptosis in AD pathology [87,88,89], further studies are urgently needed to explore DMF’s potential as a ferroptosis-targeting therapeutic in AD models.

Furthermore, some studies lacked detailed reporting of key methodological aspects such as randomization, blinding, and sample size justification, which may increase the risk of bias. Publication bias is also a consideration, as studies with positive findings are more likely to be reported. Another limitation of this review is that the literature search was restricted to two databases (PubMed and Scopus) and did not include grey literature sources. Although these databases provide broad coverage, it is possible that relevant studies indexed elsewhere may have been missed. These factors highlight the need for more rigorous and standardized approaches in future research.

In summary, the available preclinical evidence indicates that DMF consistently exerts antioxidant and anti-inflammatory effects in experimental models of AD, largely through activation of the Nrf2 pathway. These mechanisms are associated with improved neuronal function and cognitive outcomes in several studies. However, evidence for direct effects on amyloid and tau pathology remains limited and variable. The considerable heterogeneity across studies further complicates interpretation of the findings.

Building on the findings discussed above, several important questions remain regarding the neuroprotective potential of DMF in AD. Future studies should clarify optimal dosing, treatment duration, and timing of administration, as effects may vary depending on dose and disease stage. Greater standardisation of experimental protocols would also improve comparability across studies. Further research is needed to better understand the molecular mechanisms underlying DMF’s effects. While activation of the Nrf2 pathway appears central, the involvement of additional pathways, including PI3K/AKT, Wnt/β-catenin, NF-kB, and ferroptosis-related mechanisms, indicates that DMF likely acts through multiple interconnected targets. Combination therapies represent another promising area, as synergistic effects observed with compounds such as vitamin D3 and HY-021068 suggest that DMF may be more effective when used alongside agents targeting complementary pathways. In addition, potential sex- and age-dependent differences in treatment response should be explored to support more tailored therapeutic strategies. Finally, translating these findings into clinical settings remains essential. Well-designed clinical trials are needed to evaluate the safety, tolerability, and therapeutic impact of DMF in patients with AD, particularly through long-term assessments of disease progression and functional outcomes.

## 5. Conclusions

Alzheimer’s disease is a progressive neurodegenerative disorder which is characterized by Aβ accumulation, tau pathology, oxidative stress, and neuroinflammation, representing a major public health challenge. This systematic review highlights that DMF can activate the Nrf2 pathway, enhancing antioxidant and anti-inflammatory responses, and may counteract key pathological features of the disease, including Aβ and tau-related neurotoxicity. Preclinical evidence also suggests potential effects on ferroptosis and neuronal survival, although this area remains underexplored. Overall, these findings position DMF as a potentially promising candidate for further investigation as a supportive therapeutic strategy in AD, with future research needed to validate its efficacy and explore combination approaches, as there is still translational uncertainty.

## Figures and Tables

**Table 1:** SYRCLE Risk of Bias tool questions for quality assessment of in vivo studies [58].

Number	Question
Q1	Was the allocation sequence adequately generated and applied? (Selection bias)
Q2	Were the groups similar at baseline, or were they adjusted for confounders in the analysis? (Selection bias)
Q3	Was the allocation adequately concealed? (Selection bias)
Q4	Were the animals housed randomly during the experiment? (Performance bias)
Q5	Were the caregivers and/or investigators blinded from knowledge of which intervention each animal received during the experiment? (Performance bias)
Q6	Were animals selected at random for outcome assessment? (Detection bias)
Q7	Was the outcome assessor blinded? (Detection bias)
Q8	Were incomplete outcome data adequately addressed? (Attrition bias)
Q9	Are reports of the study free of selective outcome reporting? (Reporting bias)
Q10	Was the study apparently free of other problems that could result in a high risk of bias? (Other bias)

**Table 2:** Adapted questions for quality assessment of in vitro studies.

Number	Question
Q1	Was the experimental objective or hypothesis clearly stated? (Reporting bias)
Q2	Was the cell type or cell line clearly described and appropriate for the research question? (Other bias)
Q3	Was cell line authentication or contamination testing (e.g., mycoplasma testing) reported? (Other bias)
Q4	Was the number of biological replicates justified or explained? (Other bias)
Q5	Were samples (e.g., wells, cultures, plates) randomly allocated to experimental groups? (Selection bias)
Q6	Were investigators blinded to treatment groups during the experiment or data collection? (Performance bias)
Q7	Was outcome assessment performed blinded to group allocation? (Detection bias)
Q8	Were culture conditions (media, incubation, temperature, passage number, treatment timing) standardized and described? (Performance bias)
Q9	Were experiments replicated independently (biological replicates or separate experiments)? (Other bias)
Q10	Were outcome measurement methods clearly described and validated (e.g., assay type, antibodies, imaging protocol)? (Detection bias)
Q11	Were appropriate statistical analyses reported and clearly described? (Reporting bias)
Q12	Were all measured outcomes reported without evidence of selective reporting? (Reporting bias)

**Table 3:** Summary of key characteristics of the studies included in the systematic review.

Preclinical Study	Experimental Model *	DMF Dose	Dose of Other Interventions	Main Results
**In vitro studies**				
[62]	Human neuroblastoma SH-SY5Y cells	30 μM DMF	1 μM oligomer or Aβ_1–42_	Cyt-c reduction Tau phosphorylation reduction *β-catenin* and *cyclin D1* regulation Microtubule degradation prevention
[63]	Human neuroblastoma SH-SY5Y cells	10 and 30 μM DMF	1 μM oligomer or Aβ_1–42_	LDH index reduction PP2B reduction CREB phosphorylation reduction NFAT1 and BACE1 reduction No effect on APP
[64]	In vitro: Human neuroblastoma SH-SY5Y cells Ex vivo: Organotypic hippocampal slices	30 μM DMF	1 μM Aβ_1–42_	In vitro: Tau expression and phosphorylation decrease Nrf2 and Keap1 increase HO-1, GSH and MnSOD increase NF-kB decrease Ex vivo: Sustained viability Tau expression and phosphorylation decrease
[65]	Mouse neuroblastoma N2a cells Mouse microglial BV-2 cells	N2a: 14 μM DMF BV-2: 30 μM DMF	50 ng/mL LPS in BV-2	N2a: Nrf2 activation Hmox increase PKB activation No change in Ca^2+^ levels BV-2: *iNOS* and *IL-1β* reduction
[66]	Human neuroblastoma SH-SY5Y cells	0.1, 1 and 10 mM DMF	17.5 mM glucose	HO-1 and MnSOD regulation Dose-dependent Nrf2 activation GSH and ROMO-1 regulation p53 increase
[67]	Human neuroblastoma SH-SY5Y cells	10, 20 and 30 μM DMF	800 μM MGO	Nrf2 nuclear accumulation GSH increase GCLM increase MG-H1 decrease
**Mixed-methodology studies**				
[68]	In vitro: T98G astrocyte cells A1 astrocyte cells In vivo: App^NL-G-F/NL-G-F^ (App-KI) and WT mice 6 m, 11 m	In vitro: T98G: 0 and 35 μM DMF A1: 35 μM DMF In vivo: 300 mg/kg DMF	N/S	In vitro: Nrf2 activation in T98G and A1 C3 and SOCS3 reduction in A1 In vivo: *GCLM* increase Cognitive function improvement
[57]	In vitro: Primary mouse hippocampal neurons (embryonic, Nrf2^+/+^ and Nrf2^−/−^, females) In vivo: C57BL/6J mice (male) 8 w	In vitro: 0, 20, 40, 60, 80 and 100 μM DMF In vivo: 10 mg/mL DMF	2 μg/μL Aβ_1–42_ 1 μg/μL IBO	In vitro: Antioxidant action of DMF Neuron survival increase In neurons with Nrf2, ROS stable In vivo: Cognitive impairment reversal Antioxidant enzyme increase Lipid peroxidation, mitochondrial dysfunction, inflammation, and Aβ deposition suppression
[69]	In vitro: Rat mesenchymal stem cells (MSCs, male) In vivo: Wistar rats (male) adult	In vitro: 5, 10, 15, 20, 30, 40 and 50 μM DMF In vivo: 20 μM DMF	In vivo: 1 μg/μL Aβ_1–42_	In vitro: Proliferation and survival increase Nrf2 expression increase In vivo: MSC function improvement BDNF and NGF increase Bcl2 increase Bax, Caspase, and Cyt c decrease
[70]	In vitro: Microglial cells (MG) In vivo: C57BL/6, Cx3cr1^gfp/+^, and Nrf2^−/−^ mice (female) 12–16 w	In vitro: 0, 10 and 100 μM DMF In vivo: 45 mg/kg DMF	In vitro: 100 ng/mL LPS In vivo: Saline or 1 mg/kg LPS	In vitro: MG maturation marker suppression Nrf2-target gene increase Inflammatory cytokine TNF-α, IL-1β, iNOS, and interleukin reduction NF-kB activation inhibition In vivo: Cognitive disorder symptoms relief
[71]	In vitro: Mouse hippocampal HT22 cells In vivo: Transgenic APP/PS1 and WT mice (male) 6 m	In vitro: 30 μM DMF In vivo: 50 mg/kg DMF	In vitro: 40 μM HY, 5 μM Aβ_1–42_ In vivo: 5, 10 and 20 mg/kg HY	In vitro: HY loses its anti-ferroptotic and antioxidant activity in the absence of DMF In vivo: Learning and memory improvement Aβ accumulation and Aβ_1–42_ production decrease Tau phosphorylation decrease Nrf2, HO-1, NAD(P)H, and NQO1 activation GSH increase Ferroptosis reduction
**In vivo studies**				
[72]	Wistar Rats (male) 4 m	0.4% DMF	3 mg/kg STZ	Μemory disorder decrease Neurodegeneration decrease in the hippocampus IL-6 and IL-10 expression decrease in the hippocampus
[73]	Wistar Rats (male) 22 m	0.4% DMF	3 mg/kg STZ	Spatial memory improvement in aged rats CD68 reduction in aged rats Neurodegeneration prevention in young and aged rats
[74]	Wistar Rats (female) 18 m	45 mg/kg DMF	150 mg/kg/day D-Gal	Cognitive function improvement GFAP decrease AMPK/SIRT-1 and AKT/CREB/BDNF pathway activation Hippocampal adiponectin reduction BACE1, Aβ_42_, p-GSK-3β, and p-Tau decrease
[75]	NRF2-KO and NRF2-WT mice with combined amyloidosis and tauopathy 6 m, 9 m, 11 m	100 mg/kg DMF	N/S	Nrf2 expression increase COX-2, NOS-2, GFAP, IBA1, and MHCII reduction Memory function improvement
[76]	Wistar Rats (male) 4 m and 22 m	0.4% DMF	3 mg/kg STZ	Memory acquisition improvement in elderly rats Neuroinflammation reduction only in aged rats B lymphocytes, NK cells, and T CD3+CD4+CD8- and T CD3+CD4-CD8+ lymphocyte reduction in aged rats T CD3+ peripheral lymphocyte and peripheral monocyte increase in elderly rats IFN-γ and IL-6 decrease IL-10 increase in elderly rats
[77]	Wistar Rats (male) 4 m	50 mg/kg DMF	3 mg/kg STZ 2000 IU/kg Vit D3	25(OH)D3 plasma concentration increase Spatial memory improvement Tau protein level reduction Lipid peroxidation reduction GSH and IL-10 increase TNF-α and IL-6 reduction
[78]	Transgenic APPPS1–21 mice (female) 40 d, 60 d	75 mg/kg DMF	N/S	No effect on memory and learning, Aβ protein accumulation, immune system

* Abbreviations used to denote the age of animals: d = days; w = weeks; m = months.

## Data Availability

No new data were created or analyzed in this study. Data sharing is not applicable to this article. Extracted data are available from each corresponding study.

## Supplementary Materials

The following supporting information can be downloaded at: https://www.mdpi.com/article/10.3390/ijms27104227/s1.

## Abbreviations

The following abbreviations are used in this manuscript:

**Abbreviation**	**Full Term/Description**
Aβ	Amyloid-β
AD	Alzheimer’s disease
AMPK	Adenosine monophosphate-activated protein kinase
APP	Amyloid precursor protein
AREs	Antioxidant response elements
BACE1	β-Secretase 1
BDNF	Brain-derived neurotrophic factor
COX-2	Cyclooxygenase-2
CREB	cAMP response element-binding protein
Cyt c	Cytochrome c
DG	Dentate gyrus
D-Gal	D-galactose
DMF	Dimethyl fumarate
FDA	Food and Drug Administration
GCLM	Glutamate–cysteine ligase modifier subunit
GFAP	Glial fibrillary acidic protein
GPX4	Glutathione peroxidase 4
GSH	Glutathione
GSK-3β	Glycogen synthase kinase-3 beta
Hmox1/HO-1	Haem oxygenase-1
IBA1	Ionized calcium-binding adapter molecule 1
IFN-γ	Interferon-γ
IL	Interleukin
iNOS	Inducible nitric oxide synthase
LDH	Lactate dehydrogenase
LPS	Lipopolysaccharide
MAPK	Mitogen-activated protein kinase
MDA	Malondialdehyde
MG	Microglia
MG-H1	Methylglyoxal-derived hydroimidazolone 1
MGO	Methylglyoxal
MHCII	Major histocompatibility complex class II
MMF	Monomethyl fumarate
MnSOD/SOD2	Manganese superoxide dismutase
MSCs	Mesenchymal stem cells
NEHs	Nrf2–ECH homology domains
NFAT1	Nuclear factor of activated T cells 1
NF-kB	Nuclear factor kappa B
NFTs	Neurofibrillary tangles
NGF	Nerve growth factor
NOS-2	Nitric oxide synthase 2
NQO1	NAD(P)H quinone dehydrogenase 1
Nrf2	Nuclear factor erythroid 2-related factor 2
NT3	3-Nitrotyrosine
OPTN	Optineurin
OVX	Ovariectomy
PI3K	Phosphoinositide 3-kinase
PKB/AKT	Protein kinase B
PP2A	Protein phosphatase 2A
PP2B	Protein phosphatase 2B (Calcineurin)
ROMO1	Reactive oxygen species modulator 1
ROS	Reactive oxygen species
SIRT-1	Sirtuin 1
sMAFs	Small musculoaponeurotic fibrosarcoma proteins
SOCS3	Suppressor of cytokine signalling 3
STAT3	Signal transducer and activator of transcription 3
STZ	Streptozotocin
TNF-α	Tumour necrosis factor alpha
