# Supplementary material for: The Emerging Role of Dimethyl Fumarate in Alzheimer’s Disease—A Systematic Review of Available Preclinical Studies
Source: Int J Mol Sci. 2026 May 9;27(10):4227. doi: 10.3390/ijms27104227 (PMC13207082; doi:10.3390/ijms27104227)
Supplement: Supplementary file 1 [file ijms-27-04227-s001.zip › ijms-4254356- suplementary materials.pdf]

Supplementary Table S1: Full literature search strategy

Key Words	Scopus Database	PubMed Database
**Alzheimer's disease**	242.661	326.905
**Alzheimer's disease** and **Dementia**	169.475	105.733
**Alzheimer's disease** and **Incidence**	28.170	10.029
**Alzheimer's disease** and **Epidemiology**	23.895	5.674
**Alzheimer's disease** and **Pathology**	77.208	62.466
**Alzheimer's disease** and **Therapy**	77.866	45.870
**Nrf2**	37.699	51.723
**Nrf2** and **Keap1**	7.071	8.017
**Nrf2** and **ARE**	37.699	51.723
**Nrf2** and **Keap1** and **ARE**	7.071	8.017
**Nrf2** and **ROS** and **Keap1** and **ARE**	1.854	2.016
**Nrf2** and **Alzheimer's**	1.158	2.341
**DMF**	21.691	55.931
**DMF** and **Fumaria officinalis**	0	2

**DMF** and **therapies**	5.906	1.487
**DMF** and **Alzheimer's disease**	75	85
**DMF** and **Nrf2**	340	369
**DMF** and **Alzheimer's disease** and **Nrf2**	26	19
**SUM**	**45**	

Supplementary Table S2: Excluded studies

No	Authors	Title	Journal	DOI	Reason for exclusion
1	Uruno A., Yamamoto M.	The KEAP1-NRF2 system and neurodegenerative diseases	Antioxidants & Signaling	10.1089/ars.2023.0005	Not retrieved
2	Scuderi S., Ardizzone A., Paterniti I., Esposito E., Campolo M.	Antioxidant and anti-inflammatory effect of Nrf2 inducer dimethyl fumarate in neurodegenerative diseases	Antioxidants	10.3390/antiox9070630	Review
3	Majkutewicz I.	Dimethyl fumarate: a review of preclinical efficacy in models of neurodegenerative diseases	European Journal of Pharmacology	10.1016/j.ejphar.2022.175025	Review
4	Singh N., Vijayanti S., Saha L.	Targeting crosstalk between Nuclear factor (erythroid-derived 2)-like 2 and Nuclear factor kappa beta pathway by Nrf2 activator dimethyl fumarate in epileptogenesis	International Journal of Neuroscience	10.1080/00207454.2018.1441149	Review
5	Wang J., Cao Y., Lu Y., Zhu H., Zhang J., Che J., Zhuang R., Shao J.	Recent progress and applications of small molecule inhibitors of Keap1–Nrf2 axis for neurodegenerative diseases	European Journal of Medicinal Chemistry	10.1016/j.ejmech.2023.115998	Review
6	Sharkus R., Thakkar R., Kolson D., Constantinescu C.	Dimethyl Fumarate as Potential Treatment for Alzheimer's Disease: Rationale and Clinical Trial Design	Biomedicines	10.3390/biomedicines11051387	Review
7	Lee D., Gold R., Linker R.	Mechanisms of Oxidative Damage in Multiple Sclerosis and Neurodegenerative Diseases: Therapeutic Modulation via Fumaric Acid Esters	International Journal of Molecular Sciences	10.3390/ijms130911783	Review
8	Lastres-Becker I., Garcia-Yague A., Scannevin R., Casarejos M., Kugler S., Rabano A., Cuadrado A.	Repurposing the NRF2 Activator Dimethyl Fumarate as Therapy Against Synucleinopathy in Parkinson's Disease	Antioxidants & Redox Signaling	10.1089/ars.2015.6549	Not AD-related
9	Pinjala P., Tryphena K., Kulkarni A., Goswami P., Khatri D.	Dimethyl Fumarate Exerts a Neuroprotective Effect by Enhancing Mitophagy via the NRF2/BNIP3/PINK1 Axis in the MPP+ Iodide-Induced Parkinson's Disease Mice Model	Journal of Alzheimer's Disease	10.3233/ADR-230128	Not AD-related
10	Fischer K., Thewes L., Prozorovski T., Bayer M., Dietrich M., Lowin T.,	Fumarate-based drugs protect against neuroinflammation via upregulation of anti-ferroptotic pathways	Journal of Neuroinflammation	10.1186/s12974-025-03592-3	Not AD-related

	Albrecht P., Hartung H., Meuth S., Aktas O., Berndt.				
11	Mela V., Sayd Gaban A, O'Neill E, Bechet S, Walsh A., Lynch M.	The Modulatory Effects of DMF on Microglia in Aged Mice Are Sex-Specific	Cells	10.3390/cells1104729	Not AD-related
12	Pourparizi A., Vazirinia M., Pourrajab F., Nadri H., Davood A.	New synthetic derivatives of isoindoline-dione: synthesis, neuroprotection assay and impact on the expression level of oxidative stress-related genes in neuronal-like cell line	Journal of Receptors and Signal Transduction	10.1080/10799893.2023.2291559	No DMF as drug

Supplementary Table S3: Risk of bias assessment per *in vitro* study

Study	Experimental objective / hypothesis clearly stated	Cell type/line clearly described and appropriate	Cell line authentication / contamination testing	Number of biological replicates justified/explained	Random allocation of samples to groups	Blinding of investigators during experiments /data collection	Blinded outcome assessment	Culture conditions standardized/described	Independent experimental replication	Outcome measurement methods clearly described & validated	Appropriate statistical analyses clearly described	All measured outcomes reported without selective reporting	SUM
Wang et al. (2024)	yes	yes	not mentioned	no	not mentioned	no	no	yes	yes	yes	yes	yes	7
Sun et al. (2022)	yes	yes	not mentioned	no	not mentioned	not mentioned	not mentioned	yes	yes	yes	yes	yes	7
Babaei et al. (2023)	yes	yes	not mentioned	no	not mentioned	not mentioned	not mentioned	yes	yes	yes	yes	yes	7
Paraiso et al. (2018)	yes	yes	yes	no	not mentioned	not mentioned	yes	yes	yes	yes	yes	yes	9
Huang et al. (2025)	yes	yes	not mentioned	no	not mentioned	not mentioned	not mentioned	yes	yes	yes	yes	yes	7
Campolo et al. (2018)	yes	yes	not mentioned	no	not mentioned	not mentioned	not mentioned	yes	yes	yes	yes	yes	7

Koike et al. (2025)	yes	yes	not mentioned	no	not mentioned	not mentioned	not mentioned	yes	yes	yes	yes	yes	7
Lanza et al. (2023)	yes	yes	not mentioned	no	not mentioned	not mentioned	not mentioned	yes	yes	yes	yes	yes	7
Silva et al. (2020)	yes	yes	not mentioned	no	not mentioned	not mentioned	not mentioned	yes	yes	yes	yes	yes	7
Rajput et al. (2020a)	yes	yes	not mentioned	no	not mentioned	not mentioned	not mentioned	yes	yes	yes	yes	yes	7
Rajput et al. (2020b)	yes	yes	not mentioned	no	not mentioned	not mentioned	not mentioned	yes	yes	yes	yes	yes	7

Supplementary Table S4: Risk of bias assessment per *in vivo* study

Study	Allocation sequence adequately generated	Groups similar at baseline / confounder adjustment	Allocation adequately concealed	Animals housed randomly	Caregivers/investigators blinded	Animals randomly selected for outcome assessment	Outcome assessor blinded	Incomplete outcome data addressed	Free of selective outcome reporting	Free of other bias	SUM
Wang et al. (2024)	not mentioned	yes	not mentioned	not mentioned	no	not mentioned	no	yes	yes	yes	4
Sun et al. (2022)	yes	yes	not mentioned	not mentioned	not mentioned	not mentioned	not mentioned	yes	yes	yes	5
Babaei et al. (2023)	yes	yes	not mentioned	not mentioned	not mentioned	not mentioned	not mentioned	yes	yes	yes	5
Paraiso et al. (2018)	yes	yes	not mentioned	not mentioned	yes	not mentioned	yes	yes	yes	yes	7
Huang et al. (2025)	not mentioned	yes	not mentioned	not mentioned	not mentioned	not mentioned	yes	yes	yes	yes	5
Majkutewicz et al. (2016)	not mentioned	yes	not mentioned	not mentioned	not mentioned	not mentioned	yes	yes	yes	yes	5
Majkutewicz et al. (2018)	not mentioned	yes	not mentioned	not mentioned	not mentioned	not mentioned	yes	yes	yes	yes	5
Abd El-Fatah et al. (2021)	yes	yes	not mentioned	not mentioned	yes	yes	yes	yes	yes	yes	8
Rojo et al. (2018)	not mentioned	yes	not mentioned	not mentioned	yes	not mentioned	yes	yes	yes	yes	6
Wrona et al. (2022)	not mentioned	yes	not mentioned	not mentioned	not mentioned	not mentioned	not mentioned	yes	yes	yes	4
Piekarczyk et al. (2025)	not mentioned	yes	not mentioned	not mentioned	not mentioned	not mentioned	not mentioned	yes	yes	yes	4
Möhle et al. (2021)	not mentioned	yes	not mentioned	no	not mentioned	not mentioned	not mentioned	yes	yes	yes	4
